# *Cordyceps militaris* solid medium extract alleviates lipopolysaccharide-induced acute lung injury via regulating gut microbiota and metabolism

**DOI:** 10.3389/fimmu.2024.1528222

**Published:** 2025-01-20

**Authors:** Xiaoya Wang, Kang Zhang, Jingyan Zhang, Guowei Xu, Zhiting Guo, Xiaorong Lu, Chunhua Liang, Xueyan Gu, Liping Huang, Shuqi Liu, Lei Wang, Jianxi Li

**Affiliations:** Traditional Chinese Veterinary Technology Innovation Center of Gansu Province, Lanzhou Institute of Husbandry and Pharmaceutical Sciences, Chinese Academy of Agricultural Sciences, Lanzhou, Gansu, China

**Keywords:** *Cordyceps militaris* solid medium, acute lung injury, anti-inflammation, gut microbiota, metabolism

## Abstract

Acute lung injury (ALI) is a common respiratory disease, Cordycepin has been reported to reduce ALI, which is an effective component in *Cordyceps militaris* solid medium extract (CMME). Therefore, we aimed to explore the alleviating effect and mechanism of CMME on ALI. This study evaluated the effect of CMME on lipopolysaccharide (LPS)-induced ALI mice by analyzing intestinal flora and metabolomics to explore its potential mechanism. We assessed pulmonary changes, inflammation, oxidative stress, and macrophage and neutrophil activation levels, then we analyzed the gut microbiota through 16S rRNA and analyzed metabolomics profile by UPLC-QTOF/MS. The results showed that CMME treatment improved pulmonary injury, reduced inflammatory factors and oxidative stress levels, and decreased macrophage activation and neutrophil recruitment. The 16S rRNA results revealed that CMME significantly increased gut microbiota richness and diversity and reduced the abundance of *Bacteroides* compared with Mod group significantly. Metabolic analysis indicated that CMME reversed the levels of differential metabolites and may ameliorate lung injury through purine metabolism, nucleotide metabolism, and bile acid (BA) metabolism, and CMME did reverse the changes of BA metabolites in ALI mice, and BA metabolites were associated with inflammatory factors and intestinal flora. Therefore, CMME may improve lung injury by regulating intestinal flora and correcting metabolic disorders, providing new insights into its mechanism of action.

## 1 Introduction

ALI always results from various intrapulmonary (pneumonia, infection, etc.) or extrapulmonary pathogenic factors, potentially progressing to acute respiratory distress syndrome. During the development of ALI, activation of macrophages and accumulation of neutrophils (NEU) in the lungs often occur (1, 2), contributing to the occurrence of pulmonary interstitial edema and damage to alveolar epithelial cells (3, 4). And ALI is characterized by an up-regulated expression of genes involved in inflammatory responses, including tumor necrosis factor α (TNF-α), interleukins (IL-6, IL-1β) and the release of myeloperoxidase (MPO) (5). LPS, a component unique to Gram-negative bacteria, is commonly employed to induce ALI in short-term mouse models (6).

Intestinal microbiota are integral to local intestinal digestion and immunity, and recent research underscores the role of intestinal microbiota in regulating systemic physiological activities (7). These microbes may influence distal organs like the lung and brain through the gut-lung and brain-gut axes. Consequently, intestinal microbiota have emerged as a focal point in the study of distal organ diseases (8). Studies have reported that ALI mice exhibit alterations in the composition and functionality of intestinal microbiota, potentially attributed to inflammatory factors levels in the lungs (6, 9, 10). Changes in intestinal flora are pivotal in the onset and prognosis of lung diseases (11), with interconnections established through metabolites or signaling pathways (12). For instance, research by Li et al. demonstrated that total polyphenols from Nymphaea candida can ameliorate ALI by restoring intestinal flora diversity and influencing short-chain fatty acids metabolism (13). Additionally, Lu et al. found that Fuzhengjiedu can mitigate ALI by modulating intestinal flora and amino acid metabolism (14), underscoring the lung-intestinal axis as a novel avenue to explore drug efficacy in lung inflammation.

As a by-product of *Cordyceps militaris*, *Cordyceps militaris* solid medium (CMM) also contains active ingredients such as cordycepin and polysaccharide. *Cordyceps militaris*, known for its edible and medicinal properties, has long been utilized in traditional Chinese medicine to effectively tonify the lung and kidney functions. Research indicates that cordycepin exerts anti-inflammatory effects, alleviating ALI mice by inhibiting inflammatory factors and proteins on NF-κB and Nrf2/HO-1 pathway (4, 15). Yang et al. (16) further demonstrated that cordycepin suppresses MAPK and NF-κB signaling pathways to mitigate Th2 responses, suggesting anti-asthmatic potential. In addition, there have been studies on the conversion of CMM, such as Wang et al. which extracted angiotensin-I-converting enzyme from CMM (17). Our previous studies have shown that CMME modulates the NF-κB pathway to inhibit inflammation in alveolar macrophages (MH-S cells). However, the effect of CMME on LPS-induced ALI in mice remains unexplored. Thus, this study assesses the therapeutic effects of CMME on LPS-induced mice, analyzing differences in intestinal microbiota, metabolic pathways, and metabolites through 16S rRNA sequencing and metabolomics.

## 2 Materials and methods

### 2.1 Materials

LPS (*E. Coli*, O111:B4) was purchased from Sigma-Aldrich Trading Co., LTD. (Missouri, USA). CMM was obtained from Xuzhou Hongyu Technology Co., LTD. (Xuzhou, China). TNF-α, IL-1β, IL-6 ELISA Kit and BCA Protein Quantification Kit were all purchased from Beyotime Biotechnology Co., LTD. (Shanghai, China). Superoxide Dismutase (SOD), malondialdehyde (MDA), catalase (CAT) and MPO kits were all purchased from Nanjing Jiancheng Biological Engineering Co., LTD. (Nanjing, China). RNA extraction kit, reverse transcription kit and SYBR fluorescent dye kit were purchased from AG Biotechnology Co., LTD. (Hangzhou, China).

### 2.2Components of CMME

CMM was treated with ultrasonic-enzyme method (KQ-600DE, China), the extraction conditions were 3.1% cellulase, 60 min, 59°C, 1:42 (g/mL), supernatant was obtained by centrifugation, and CMME was freeze-dried to detect the composition of extracts. Appropriate amount of evenly mixed samples was placed in a 2 mL centrifuge tube and subjected to centrifugation at 4°C (12000 rpm, 10 min). The supernatant was passed through 0.22 μm microporous filtration membrane and then added into 100 μg/mL internal standard solution (2-chloroalanine) to detect on the machine. The full spectrum of the samples was analyzed by LC-MS (Thermo Fisher Scientific, USA) on a C18 column (2.1 mm*100 mm, 1.8 μm).

### 2.3 Experimental animals and design

A total of 72 SPF Balb/c male mice, aged 6-8 weeks, were purchased from Lanzhou Veterinary Research Institute, Chinese Academy of Agricultural Sciences. The mice were housed under consistent conditions, with a temperature maintained at 22°C ± 1°C, and a 12-hour light/dark cycle, and they had free access to food and water. The mice were randomly allocated into six groups: control group (Con), LPS group (Mod), cordycepin group (COR, 40 mg/kg), low CMME group (CMME-L, 200 mg/kg), Mid CMME group (CMME-M, 400 mg/kg), and high CMME group (CMME-H, 800 mg/kg). Following a 7-day acclimation period, all groups were given LPS by nasal drops (3 mg/kg) except the Con group. After 24 hours, the Con and Mod groups were given normal saline via gavage, while the treatment groups received their respective doses of CMME for three days. At the conclusion of the experiment, a 12-hour fasting period was implemented for the mice prior to sacrifice. Blood and bronchoalveolar Lavage Fluid (BALF) were collected, while the right upper lobe of the lung was fixed in 4% paraformaldehyde, remaining lung tissues were stored at -80°C. Cecum contents were collected under sterile conditions and frozen at -80°C after quick freezing with liquid nitrogen.

### 2.4 Lung wet-dry weight ratio

After the experiment, lung tissues were excised, blood stains and connective tissue were removed, then weighed to determine their wet weight. Subsequently, the lung samples were placed in a constant temperature drying oven at 60°C for 24 hours. After this period, they were weighed again to record their dry weight. The lung W/D ratio was calculated to assess the extent of pulmonary edema.

### 2.5 Hematoxylin-eosin staining

Lung tissues were embedded in paraffin blocks and subsequently stained with HE staining to facilitate histological examination. The sections were then observed under an optical microscope. The evaluation process involved the random selection of three fields within each group for assessment purposes. The measurement of pulmonary septum thickness was conducted to assess the extent of lung injury.

### 2.6 Blood routine

The whole blood of mice was collected and tested with the three-classification blood routine apparatus to count the number of white blood cells (WBC), monocytes (MON), NEU, lymphocytes (LYM), hemoglobin (HGB), platelets (PLT) and red blood cells (RBC).

### 2.7 Detection of inflammatory factors and oxidation indexes

The TNF-α, IL-1β and IL-6 levels in serum, lung tissue and BALF were detected, and SOD, MDA, CAT and MPO in lung tissue were determined in strict accordance with kit instructions.

### 2.8 The mRNA expression of inflammatory factors in lung tissue

Total RNA was extracted from lung tissue using an RNA extraction kit, and reverse transcription and fluorescent quantitative PCR were performed according to the kit steps to determine the relative mRNA expression levels of TNF-α, IL-1β and IL-6 in lung tissue. Primers are shown in **Table 1**.

**Table 1:** Sequence of primes for RT-PCR.

Gene	Primer sequences (5’-3’)	Genbank accession no	Length (bp)
TNF-α	F: GGACTAGCCAGGAGGGAGAACAG	NM_214022	103 bp
	R: GCCAGTGAGTGAAAGGGACAGAAC		
IL-1β	F: CCTGGGCTGTCCTGATGAGAG	NM_008361.4	131 bp
	R: TCCACGGGAAAGACACAGGTA		
IL-6	F: CTTCTTGGGACTGATGCTGGTGAC	NM_031168.2	91 bp
	R: TCTGTTGGGAGTGGTATCCTCTGTG		
GAPDH	F: CTTCTCCTGCAGCCTCGT	NM_001411843.1	139 bp
	R: TCATCCACCTCCCCACAGTA		

### 2.9 IF staining

The distribution of neutrophils and macrophages in lung tissue was analyzed by labeling neutrophils with CD11b and Ly6G, and macrophages with CD68 and F4/80. The method was modified according to Gong (18).

### 2.10 Analysis of gut microbiota structure

DNA was extracted from six cecum content samples in each group, followed by amplification of the highly variable V3-V4 region of the bacterial 16S rRNA gene using PCR. The specific primers utilized for PCR were 341F: ACTCCTACGGGAGGCAGCA and 806R: GGACTACHVGGGTWTCTAAT (19). The constructed libraries were first inspected, and qualified libraries were sequenced using the Illumina NovaSeq 6000 (Illumina, USA). High-quality sequences were clustered, OTUs were classified, and species classification was determined based on the sequence composition of the features. Subsequent analyses included alpha diversity, beta diversity, and species distribution.

### 2.11 Non-targeted metabolomics analysis

Six plasma samples were taken from Con, LPS and CMME-M groups, respectively. Metabomic analysis was conducted by Vanquish UHPLC (Thermo Fisher Scientific, USA) and Waters ACQUITY UPLC BEH Amide (2.1 mm * 50 mm, 1.7 μm) liquid chromatographic column. Differential metabolic pathways and metabolites were screened among the groups.

### 2.12 Targeted BA metabolism detection

Sample processing is consistent with non-targeted metabolomics processing, supernatant is taken into the sample vial for UHPLC-MS/MS analysis. The target compounds were separated by Waters ACQUITY UPLC BEH C18 (2.1 mm * 150 mm, 1.7 μm) liquid chromatographic column. The concentration of target metabolites was quantitatively determined by reference to the internal standard calibration curve.

### 2.13 Statistical analysis

All data were statistically analyzed using SPSS 26.0 statistical software, and the results were expressed as mean ± SD. One-way analysis of variance was performed between groups, and P<0.05 was considered statistically significant.

## 3 Results

### 3.1 The main components of CMME

We further analyzed the main components in CMME, using the comparative analysis of mzCloud and mzValut local databases according to secondary mass spectrometry. ESI ± chromatogram is shown in **Supplementary Figure S1**. We found that the main components of CMME include cordycepin, cinnamic acid, chenylacetylene, PEG-4, L-Norleucine2-Hydroxycinnamic acid, 2-Hydroxycinnamic acid, Valine, 5’-Deoxy-5’-[4-(ethoxycarbonyl)-1-piperidinyl] uridine, trans-3-Indoleacrylic acid and choline. The specific components were shown in **Supplementary Table S1**.

### 3.2 CMME alleviates lung tissue damage in ALI mice

**Figure 1:**
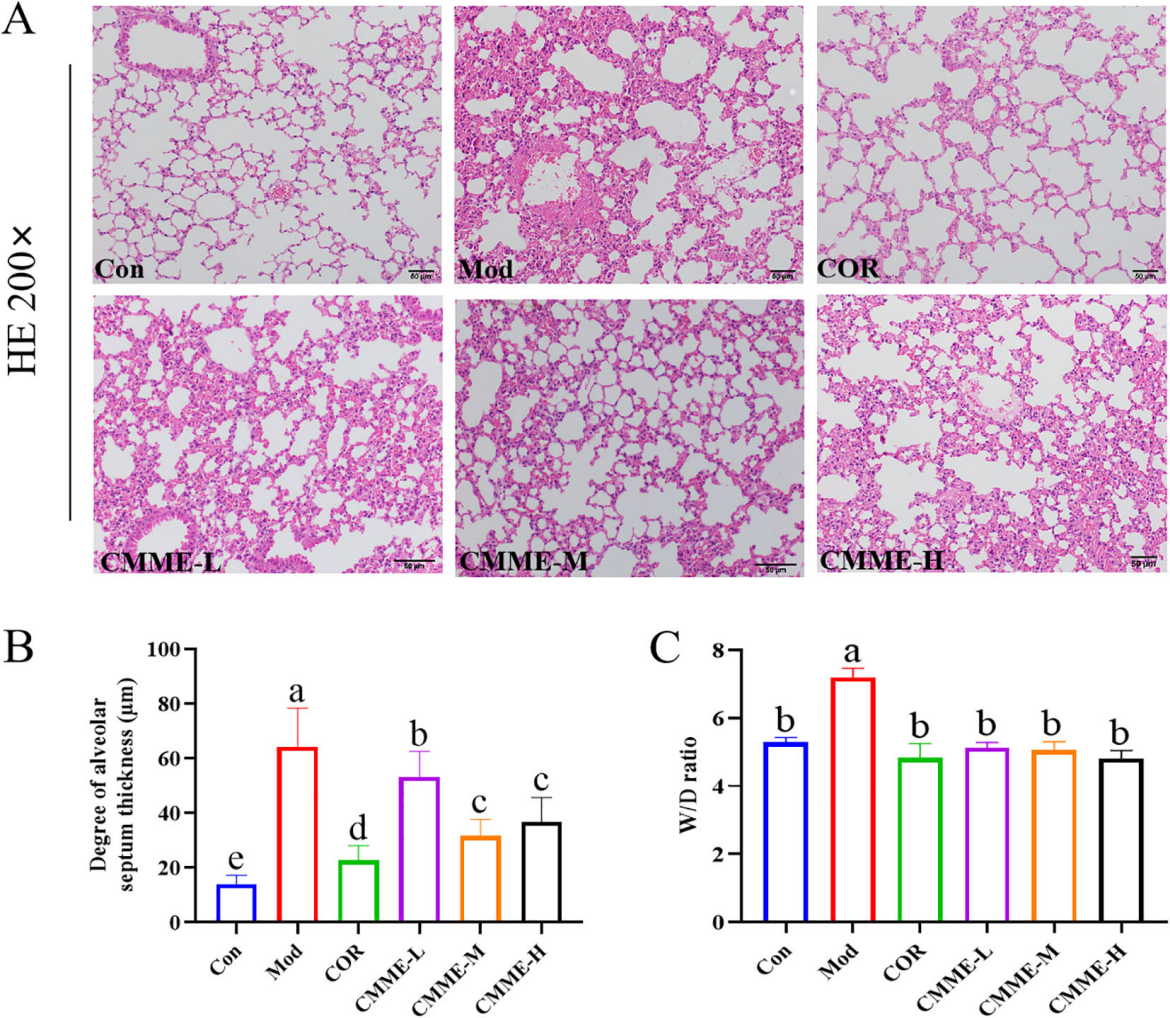
CMME alleviates edema and pathological changes of ALI mice lung tissue. **(A)** HE staining was used to observe the pathological changes of ALI mice lung tissue. **(B)** W/D ratio of lung tissue. **(C)** alveolar septal thickness. Different lowercase letters indicate significant differences, P<0.05.

The pathological sections of lung tissue, along with the statistical results regarding pulmonary septum thickness for each group, are presented in **Figure 1**. The alveolar structure in Con group was complete, without septal edema and a minimal presence of inflammatory cell infiltration. The alveolar septal thickness of Mod group was significantly greater than that of Con group (P<0.05). The lung tissue structure of mice in the CMME group was improved compared to that in the Model group significantly, and both the degree of alveolar septal thickness in CMME groups were significantly reduced (P<0.05) (**Figure 1B**).

### 3.3 CMME reduces lung W/D ratio

The lung W/D ratio is a valuable indicator for evaluating the degree of lung tissue edema in mice. As shown in **Figure 1C**, exposure to LPS markedly induces lung tissue edema compared to the Con group. However, CMME significantly reduces the extent of lung tissue edema, demonstrating its efficacy (P<0.05).

### 3.4 CMME reduces the level of inflammatory factors

**Figure 2:**
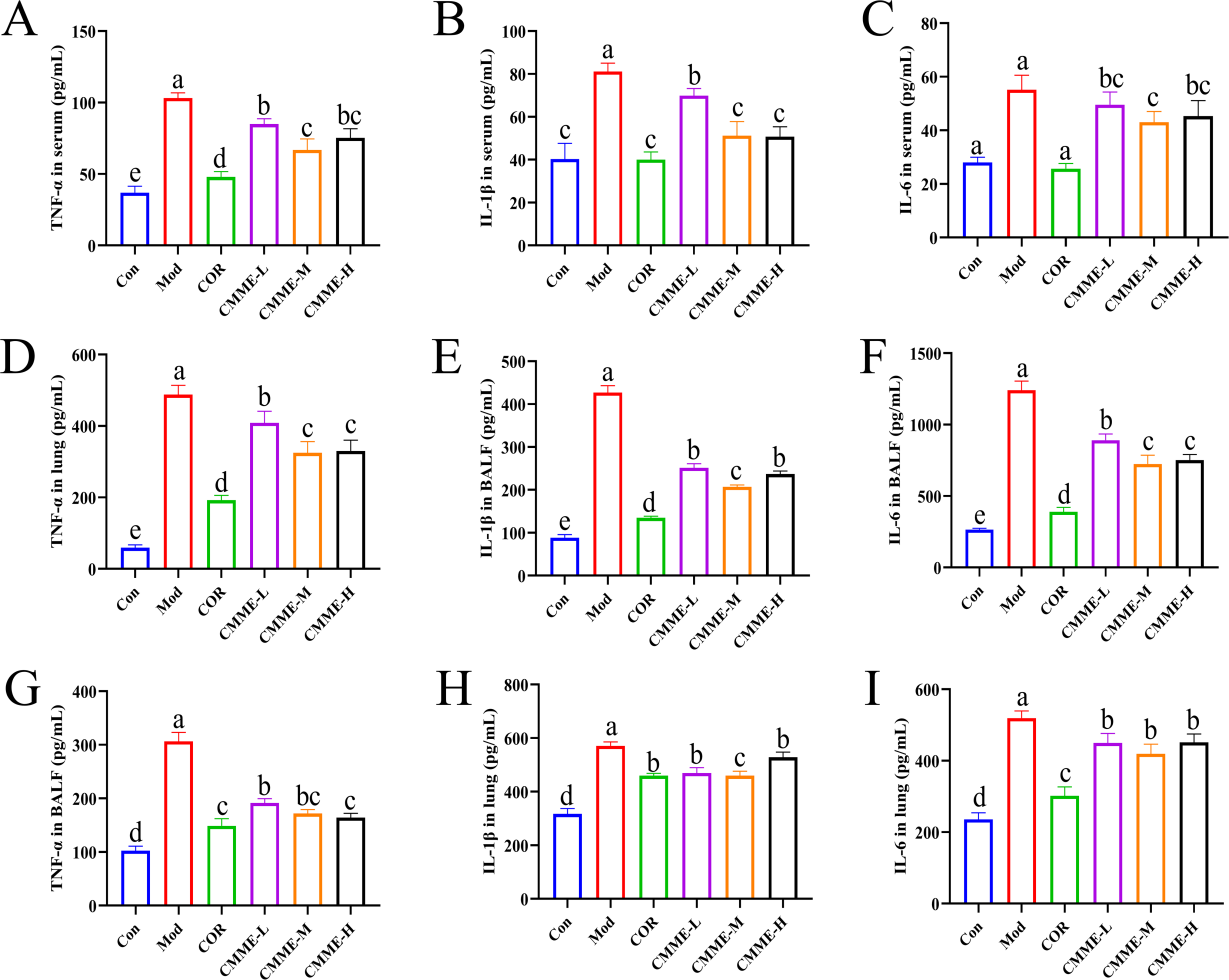
CMME reduced the levels of TNF-α, IL-1β, and IL-6 in serum, BALF, and lung tissue of ALI mice. **(A-C)** inflammatory factors levels in serum. **(D–F)** inflammatory factors levels in BALF. **(G-I)** inflammatory factors levels in lung. Different lowercase letters indicate significant differences, P<0.05.

As displayed in **Figure 2**, LPS treatment resulted in a significant increase in the levels of TNF-α, IL-1β, and IL-6 in serum, tissue, and BALF (P<0.05). All drug treatment groups demonstrated a significant reduction in the production of inflammatory factor. In the CMME group, the CMME-M showed the best performance and a more pronounced effect on serum IL-1β, which were comparable to those of the COR group. Additionally, CMME had a weak effect on inflammatory factors in lung tissue but a strong effect on BALF.

### 3.5 CMME down-regulates mRNA expression of inflammatory factors

**Figure 3:**
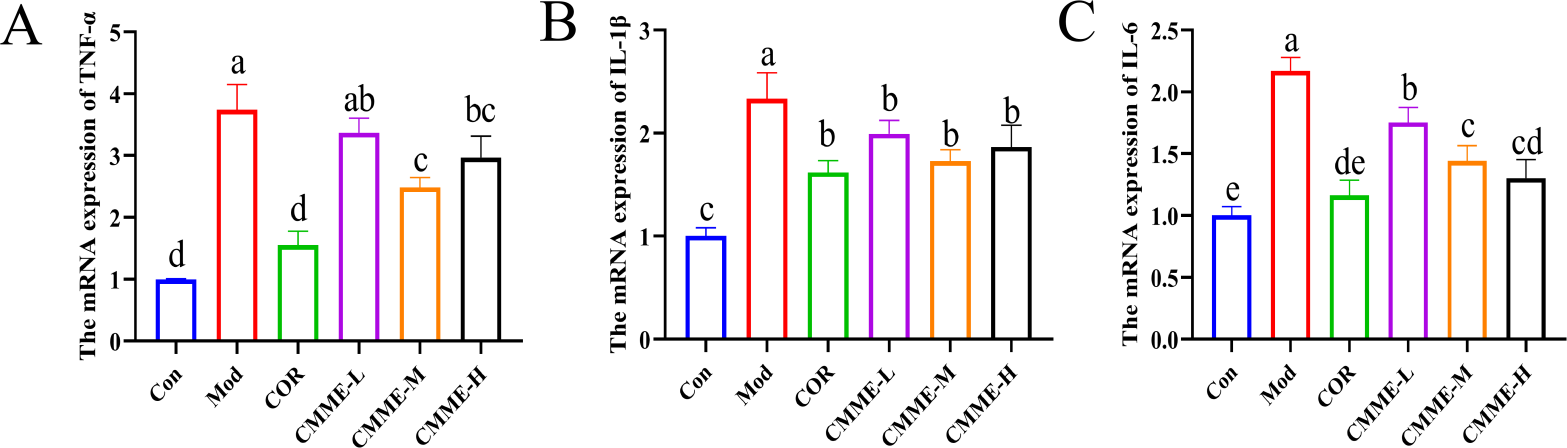
CMME reduced the relative expression levels of TNF-α, IL-1β, and IL-6 in lung tissue of ALI mice. **(A)** TNF-α. **(B)** IL-1β. **(C)** IL-6. Different lowercase letters indicate significant differences, P<0.05.

The mRNA expression levels of TNF-α, IL-1β, and IL-6 in lung tissue of each group are compared in **Figure 3**. LPS significantly increased the mRNA expression levels of TNF-α, IL-1β, and IL-6 (P<0.05), and all drug treatment groups showed a significant inhibitory effect. The mRNA expression of TNF-α and IL-1β in the CMME-M group was lower than in CMME-L and CMME-H groups, while the inhibitory effect of CMME-H on IL-6 mRNA was the strongest, although there was no significant difference between the CMME-M group (P>0.05).

### 3.6 CMME inhibits oxidative stress in ALI mice

**Figure 4:**
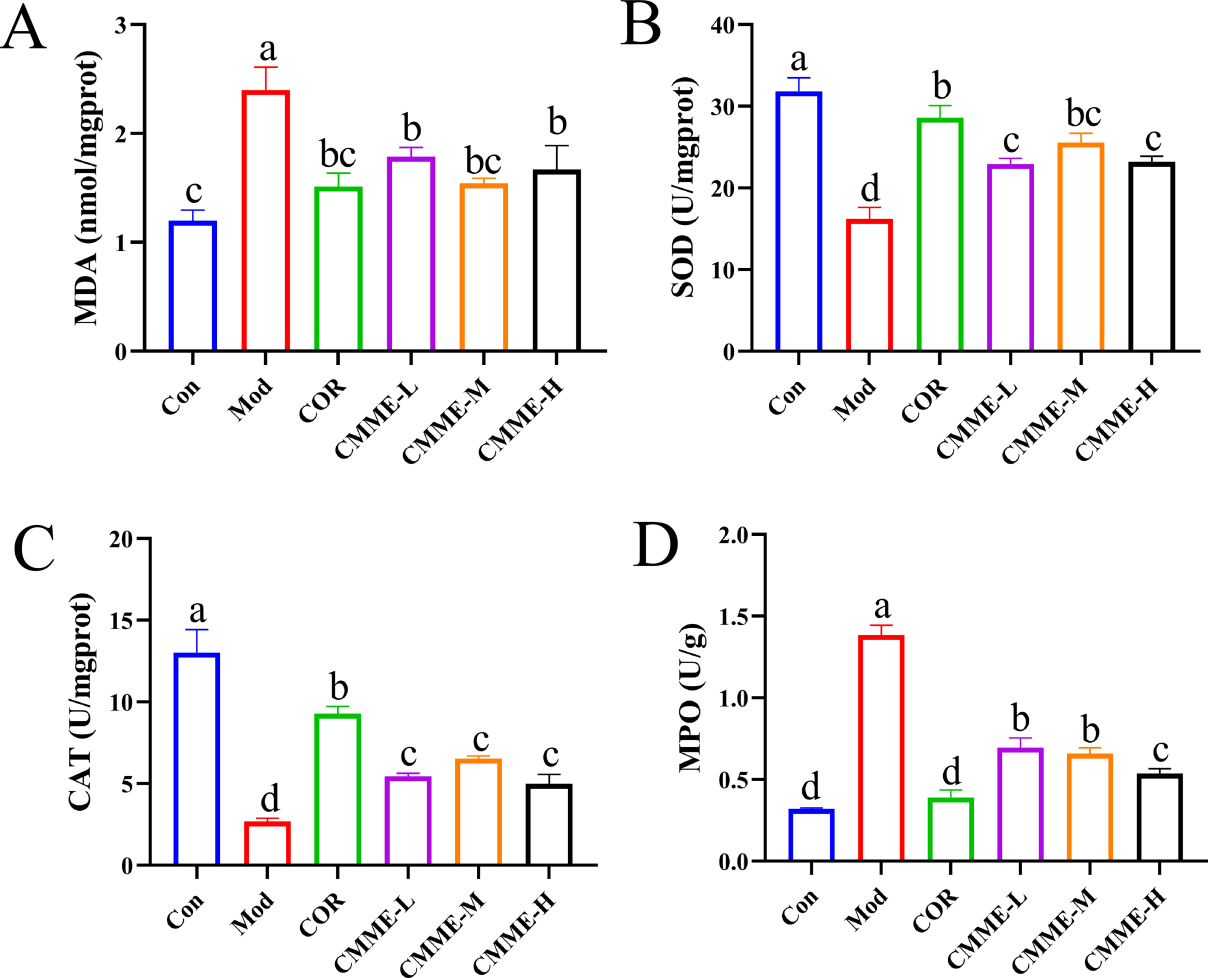
CMME reduced oxidative stress levels in lung tissue of lung tissues. **(A)** MDA. **(B)** SOD. **(C)** CAT. **(D)** MPO. Different lowercase letters indicate significant differences, P<0.05.

To explore whether CMME can mitigate the oxidative stress levels in lung, relevant oxidative stress indexes were detected, and the results are displayed (**Figure 4**). The MDA and MPO levels in LPS-induced lung tissues of mice were significantly higher than those in the Con group (P<0.05), and the contents of MDA and MPO in the COR group were reduced to levels comparable to those observed in the Con group with no significant difference noted (P>0.05). The CMME-M group demonstrated the best performance in reducing MDA content, with no significant difference between CMME and the Con group (P>0.05). The CMME-H group showed the most effective performance in reducing MPO content. SOD and CAT levels in LPS-induced lung tissues were significantly lower than those in the Con group (P<0.05). In contrast, the CMME group exhibited a significant increase in SOD and CAT contents (P<0.05), with the CMME-M group demonstrating the most pronounced effects.

### 3.7 Blood routine

**Figure 5:**
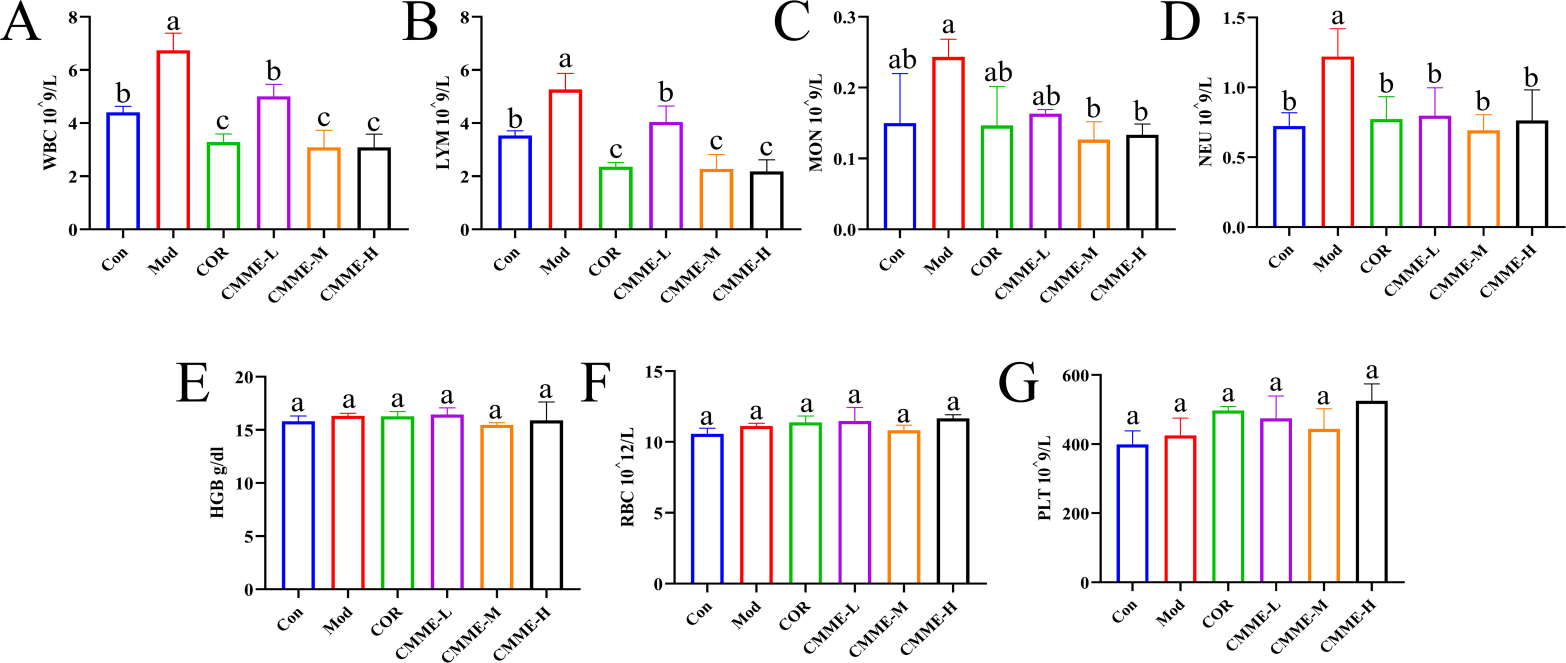
Blood routine analysis. **(A)** WBC. **(B)** LYM. **(C)** MON. **(D)** NEU. **(E)** HGB. **(F)** RBC. **(G)** PLT. Different lowercase letters indicate significant differences, P<0.05.

Blood routine results for mice in each group showed that WBC, LYM, NEU, and MON had significant changes in the blood of LPS-induced mice (P<0.05), which were higher than that in the Con group, indicating inflammation (**Figure 5**). Among these, WBC and LYM cells showed the most significant changes, with a notable reduction in their numbers observed in the CMME treatment group (P<0.05), indicating that CMME treatment group effectively mitigated inflammation in mice.

### 3.8 CMME inhibits the expression of inflammatory cells

**Figure 6:**
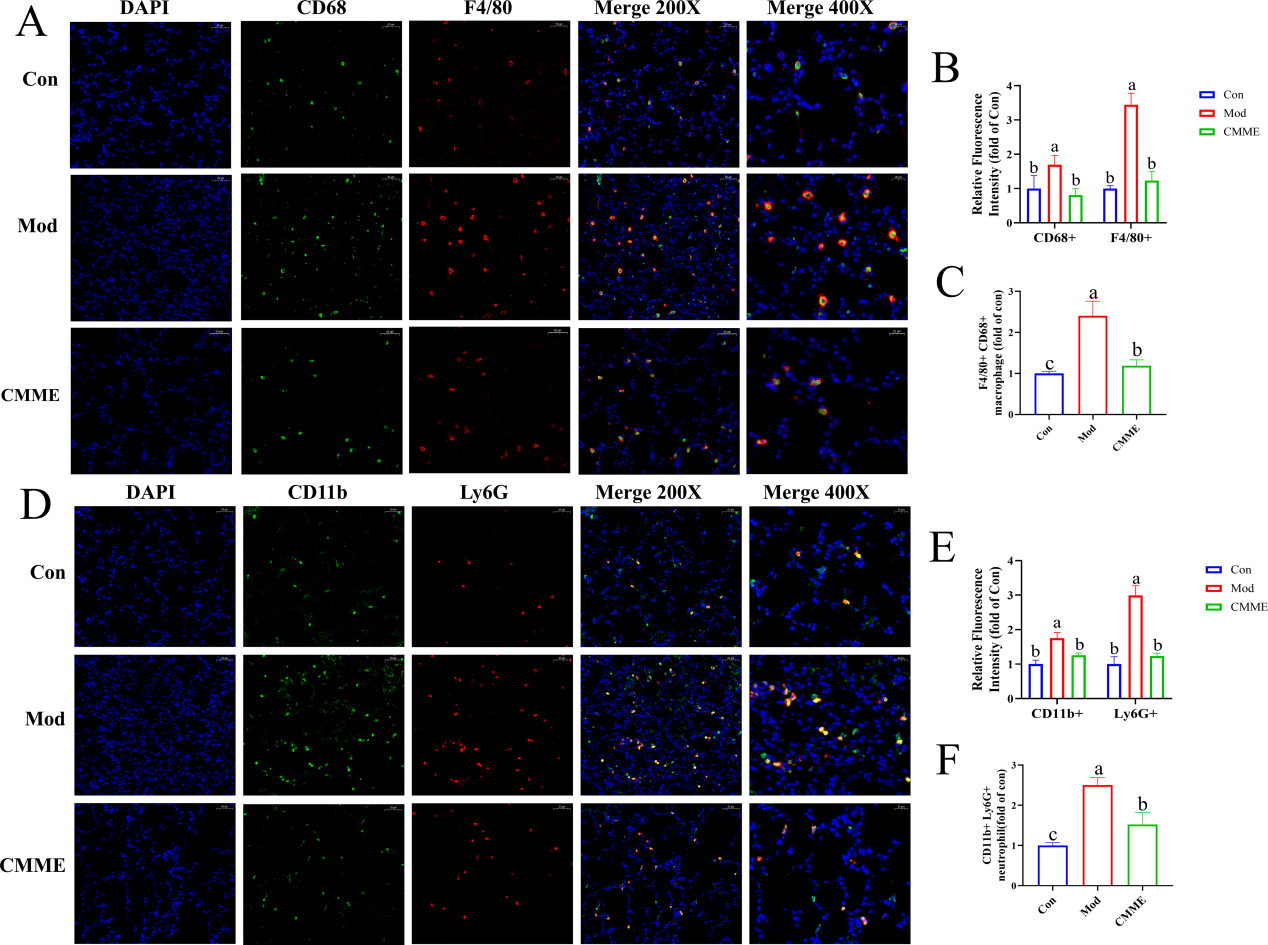
**(A)** F4/80 and CD68. **(B)** Relative fluorescence intensity of F4/80 and CD68. **(C)** F4/80 and CD68-labeled macrophage. **(D)** Ly6G and CD11b. **(E)** Relative fluorescence intensity of Ly6G and CD11b. **(F)** Ly6G and CD11b-labeled neutrophil.

The labeling results of macrophages and NEU were shown in **Figure 6**, and statistical analysis of fluorescence intensity showed CD68 and F4/80 in the Mod group labeled macrophages were prominently observed, indicating that LPS caused the activation of macrophages in lung tissue. However, in CMME group, the expression of CD68 and F4/80 was significantly reduced (P<0.05), which decreased the activation degree of macrophages. In addition, CD11b and Ly6G-labeled neutrophils were significantly elevated in the Mod group, whereas the expression levels of CD11b and Ly6G were notably decreased in the CMME group (P<0.05), indicating that neutrophil recruitment occurs in ALI, and CMME can inhibit the recruitment of neutrophils.

### 3.9 CMME modulated the microbiota of ALI mice

#### 3.9.1 OTU level

**Figure 7:**
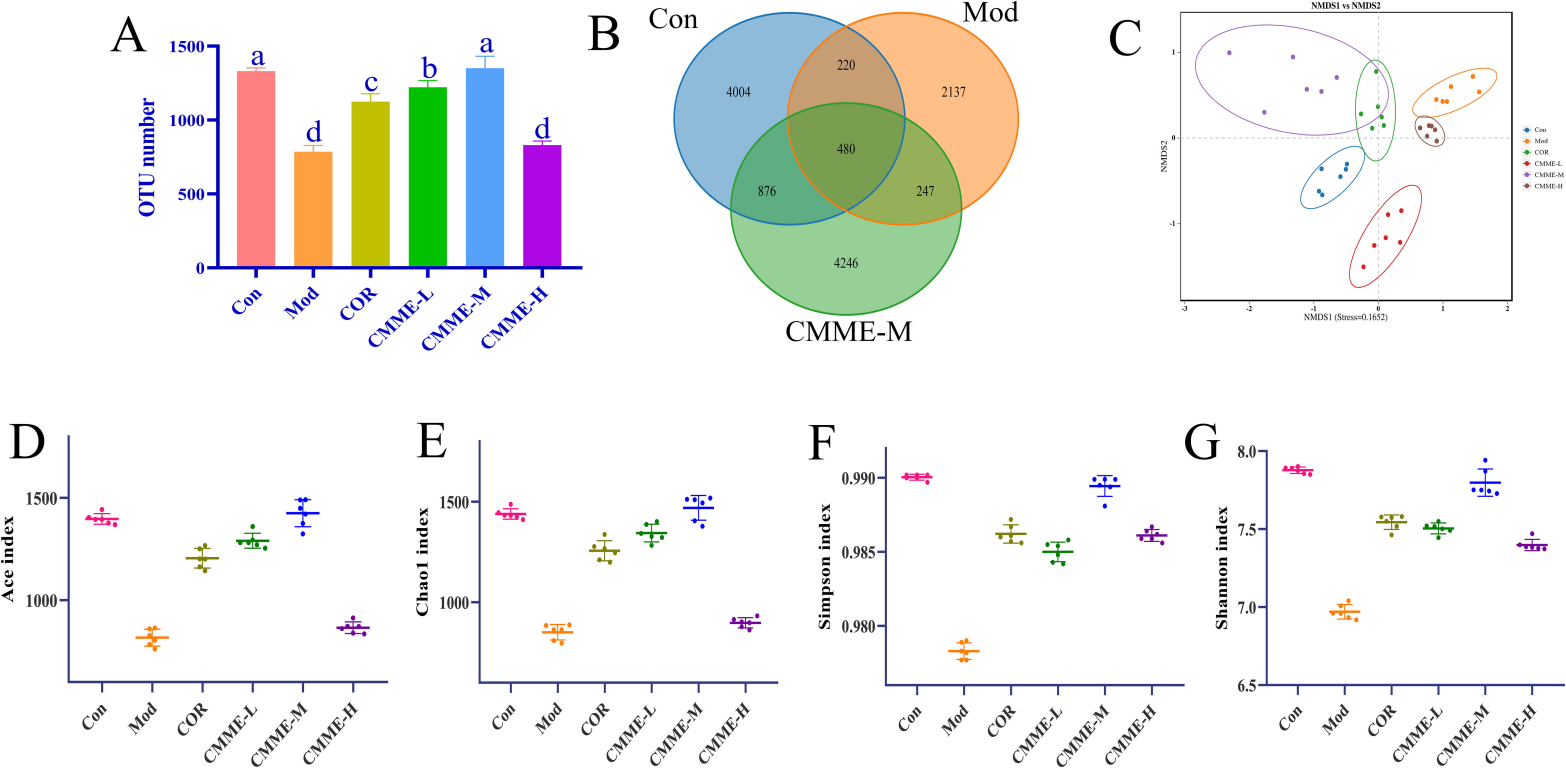
Microbiota analysis on OTU level. **(A)** OTU number. **(B)** Veen chart. **(C)** NMDS diagram. **(D)** ACE. **(E)** Chao 1. **(F)** Shannon. **(G)** Simpson. Different lowercase letters indicate significant differences, P<0.05.

Analysis of intestinal flora by 16S rRNA technology were shown in **Figure 7**, which revealed a significantly lower number of OTUs in Mod group compared to the Con group (P<0.05), while the CMME group exhibited a higher OTU number relative to the Mod group. Analysis of α diversity (Ace, Chao1, Simpson, Shannon) indicated that LPS treatment significantly reduced intestinal flora diversity indices compared to Con group (P<0.05), with α diversity indices of the COR and CMME groups higher than those of Mod group, and the CMME-M showing even greater improvement than COR. These results indicate that CMME can reverse the reduction in diversity and evenness observed in the Mod groups. β diversity analysis (NMDS) showed that the Mod group exhibited the greatest distance from other groups, suggesting a marked difference in microbial composition between the mice in Mod and Con group, while samples from the CMME and COR groups were closer to the Con group, indicating that CMME can ameliorate LPS-induced microbial disorder. The CMME-H group showed the least improvement in intestinal flora, while the CMME-M group demonstrated the greatest improvement.

#### 3.9.2 Phylum level

**Figure 8:**
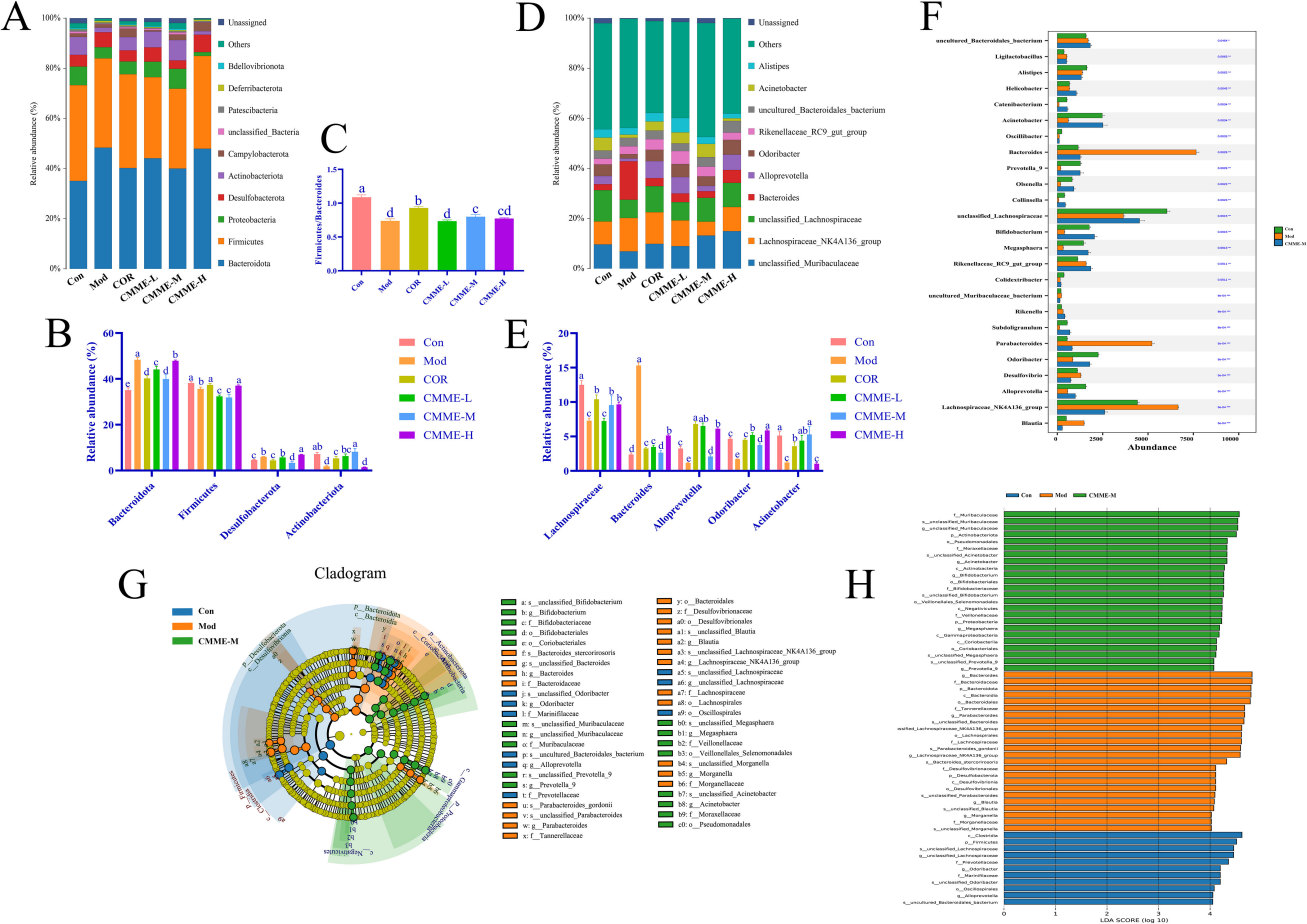
Intestinal flora structure analysis and LEfSe analysis. **(A)**Top ten bacteria on phylum. **(B)** Firmicutes/Bacteroidetes ratio. **(C)** Histogram of relative abundance of bacteria on phylum level. **(D)** Top ten bacteria on genus. **(E)** Histogram of relative abundance of bacteria on genus level. **(F)** Kruskal-Wallis on genus. **(G)** LEfSe analysis. **(H)** Each group of bacteria had an LDA score of at least 4. Different lowercase letters indicate significant differences, P<0.05.

The composition of phylum level flora in each group is shown in the **Figure 8**, the intestinal flora in all groups was predominantly composed of *Firmicutes* and *Bacteroidetes*. The abundance of *Bacteroidetes* in the Mod group was 48.30%, which was significantly higher than that in Con group (35.1%) (P<0.05), while the abundance of *Bacteroidetes* in all treatment groups was significantly lower than that found in the Mod group (P<0.05). The *Firmicutes/Bacteroidetes* ratios, which typically reflect the major flora composition of intestinal flora, were 1.09, 0.74, 0.93, 0.73, 0.80, and 0.77 in each group. Additionally, the abundance of *Actinobacteria* in the Mod group was found to be reduced compared to the Con group, while it exhibited an increase in other treatment groups relative to the Mod group. Furthermore, LPS treatment led to a proliferation of *Desulfobacterota* in the mice’s intestines, which was reversed by CMME intervention. In summary, CMME improves microflora disturbances and structural changes at the phylum level induced by LPS.

#### 3.9.3 Genus level

In terms of sample structure and distribution, further analysis of the flora (**Figure 6**) revealed the top 10 species in each group, including *Lachnospiraceae*, *Acinetobacter*, *Bacteroides*, *Alloprevotella*, and *Odoribacter*, which were predominant across all groups. In the Con group, *Lachnospiraceae* and *Acinetobacter* were the dominant bacteria. Importantly, the proportion of *Bacteroides* in the Mod group was significantly greater than that in other groups (P<0.05). **Figure 8** illustrates that *Lachnospiraceae* were notably reduced in the model group. Conversely, the abundances of *Acinetobacter*, *Rikenella* and *Odoribacter* were significantly enhanced in the CMME treatment group (P<0.05). CMME-M exhibited a significantly higher efficacy in increasing the abundance of *Acinetobacter* and *Bacteroides* compared to the COR group (P<0.05). Based on the performance of various CMME dosages, differences in microbial community abundance between CMME-M, Con, and Mod samples were selected for further study. Metas analysis highlighted significant differences between the top 25 genera at the taxonomic level, as shown in **Figure 6F** Kruskal-Wallis, showing that CMME was able to alter the gut microbiome of LPS-induced ALI mice and distinguish the microbiome composition of the three groups of mice. *Bacteroides*, unclassified_*Lachnospiraceae*, *Parabacteroides*, *Alloprevotella*, *Megasphaera* and *Bifidobacterium* in ALI mice were significantly changed than normal mice, which was reversed by CMME.

#### 3.9.4 LEfSe analysis and prediction

LEfSe analysis identified key microbial players in each group. **Figures 8G, H** presents the Cladogram and LDA diagrams. Species with LDA scores exceeding 4 in each group were analyzed as representative species. In the Con group, predominantly enriched microorganisms included c_*Clostridia*, p_*Firmicutes*, s_*unclassified_Lachnospiraceae*, and g_*unclassified Lachnospiraceae*. In contrast, in the Mod group, higher proportions were observed in g_*Bacteroides*, f_*Bacteroidaceae*, p_*Bacteroidota*, s_*unclassified_Parabacteroides*, p_*Actinobacteria*, p_*Proteobacteria*, f_*Desulfovibrionaceae*, and g_*Blautia*. Notably, the CMME treatment group exhibited substantial enrichment in g_*unclassified_Muribaculaceae* and p_*Actinobacteriota*, g_*Acinetobacter*, g_*Bifidobacterium*.

### 3.10 Effects of CMME on plasma metabolism profile

**Figure 9:**
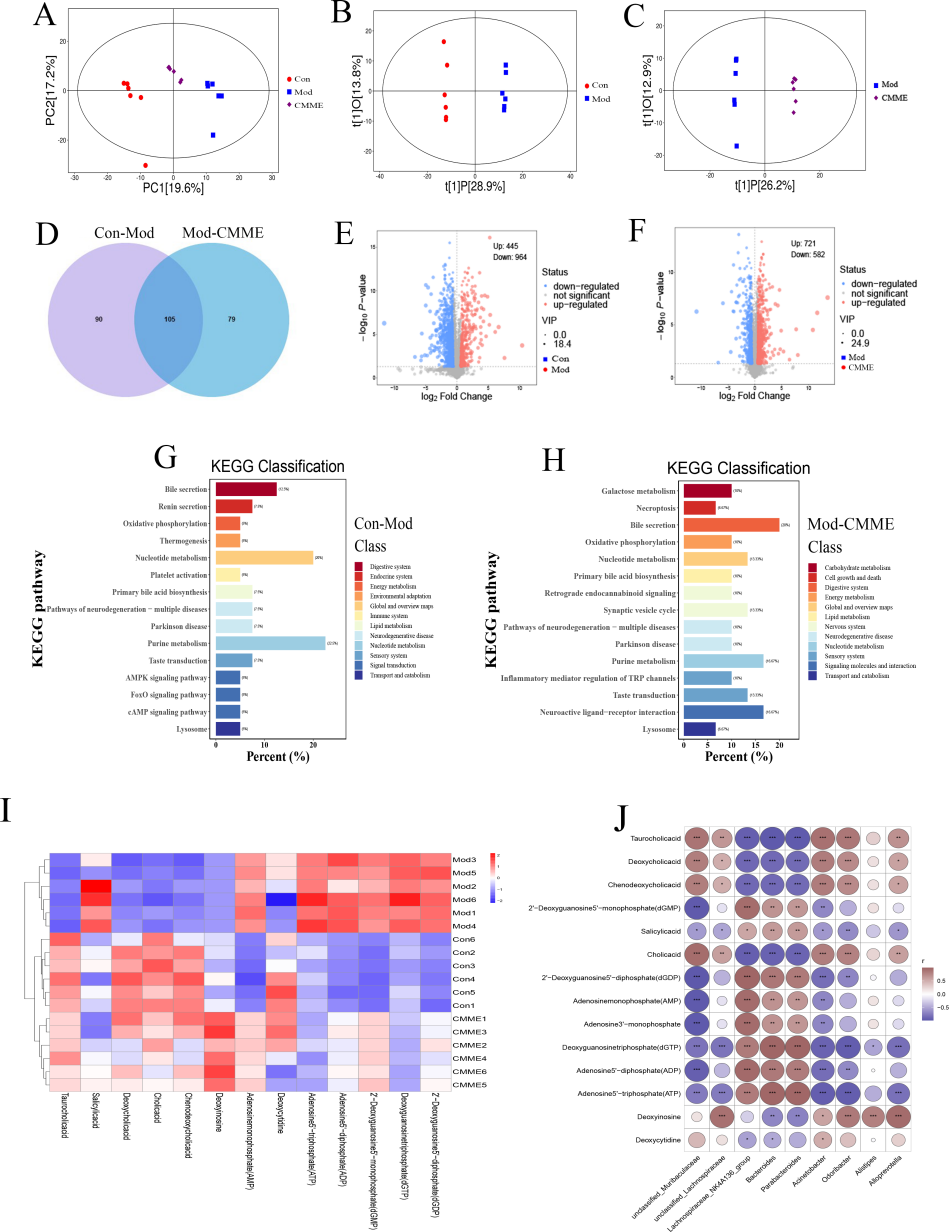
Untargeted metabolomics analysis. **(A)** PCA diagram. **(B)** OPLS-DA, Con-Mod. **(C)** OPLS-DA, Mod-CMME. **(D)** Veen chart of differential metabolites. **(E)** Volcanic map, Con-Mod. **(F)** Volcano map, Mod-CMME. **(G)** KEGG pathway analysis, Con-Mod. **(H)** KEGG pathway analysis, Mod-CMME. **(I)** Heat maps of differential metabolites on the three pathways. **(J)** Correlation analysis of metabolites in three different metabolic pathways and different bacteria genera.

The metabolic levels of plasma samples were analyzed by UPLC-QTOF/MS untargeted metabolomics. From the principal component analysis (PCA) diagram shown in **Figure 9A**, it is evident that the metabolic spectrum distribution of samples within each group is distinct, with samples intra-group showing clear clustering, and those inter-group exhibiting significant dispersion, suggesting high intra-group similarity and low inter-group similarity. Additionally, PLS-DA and one-way variance, combined with the OPLS-DA model, were used to further determine group separation, and VIP and P-values were used for analysis. VIP>1.5 and P<0.05 were established as criteria to identify the differences of metabolites among all groups. The Venn diagram (**Figure 9D**) indicated that 195 metabolites differed between the Con and Mod groups, with 69 up-regulated and 126 down-regulated. Comparatively, 184 metabolites varied between the CMME and model groups, with 95 up-regulated, 89 down-regulated, and 105 overlapping metabolites, highlighting CMME’s potential impact on plasma metabolic profiles. Pathway enrichment analysis via the MetaboAnalyst online platform further elucidated CMME’s effect on responsive metabolites. The enrichment of KEGG pathways revealed that the major repeated differential enrichment pathways between Con-Mod and Mod-CMME were nucleotide, BA and purine metabolic pathways. As shown in the heat map **Figure 9I**, the analysis of the metabolites enriched in the BA metabolic pathway showed that CMME reversed the down-regulation of taurocholic acid (TCA), cholic acid (CA), deoxycholic acid (DCA) and chenodeoxycholic acid (CDCA). Analysis of differential metabolites enriched in purine metabolism and nucleotide metabolism found that, compared with the Con group, The Mod group causes the significant changes in deoxycytidine, deoxyinosine, adenosine5’-diphosphate (ADP), 2’-Deoxyguanosine-5’-diphosphate (dGDP), 2’-Deoxyguanosine5’-monophosphate (dGMP), adenosine monophosphate (AMP), deoxyguanosine triphosphate (dGTP) and adenosine5’-triphosphate (ATP) metabolites. CMME can significantly reverse the upregulation of ATP, ADP, dGTP and the downregulation of deoxycytidine, deoxyinosine caused by LPS. And the correlation analysis of differential metabolites enriched in the above three major metabolic pathways and differential bacteria genera were carried out and shown in **Figure 9J**. There was no strong correlation between differential metabolites and unclassfied_*Lachnospiraceae* and *Alistipes*, but there was strong correlation with other bacteria genera. This suggests that intestinal flora, BA metabolism, purine metabolism and amino acid metabolism may be involved in the remission of ALI under the intervention of CMME.

### 3.11 Targeted BA metabolomics and correlation analysis

**Figure 10:**
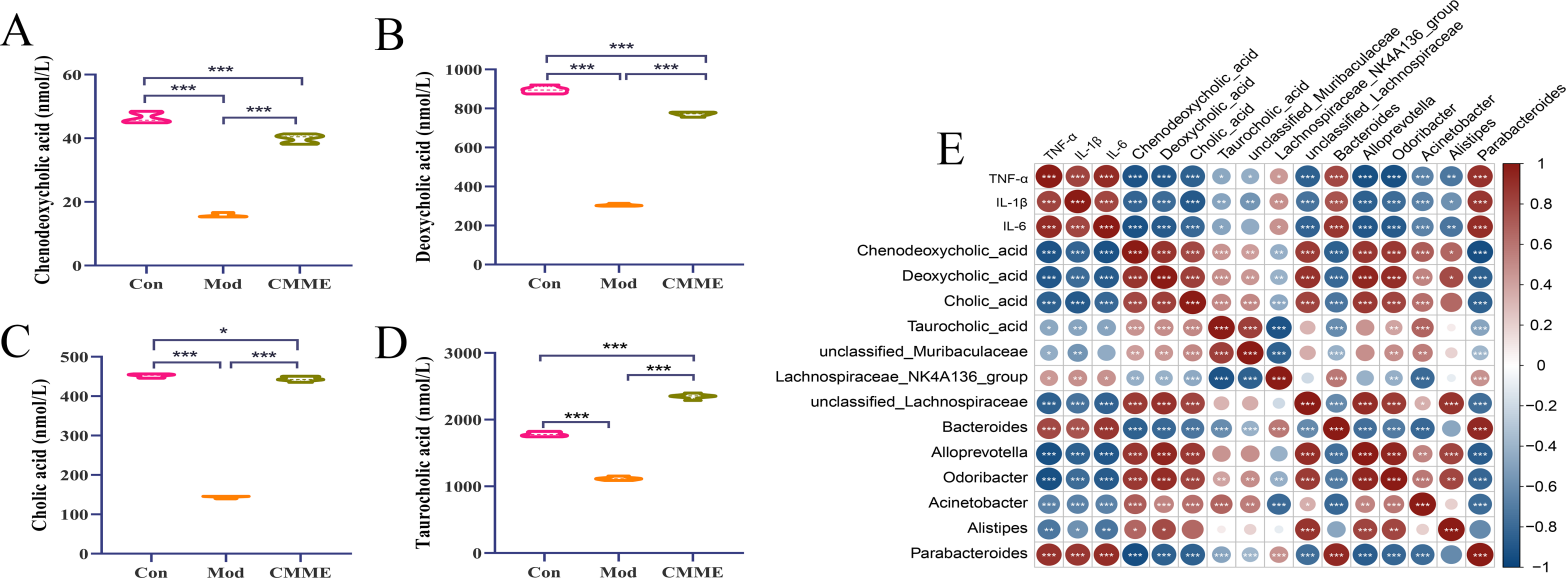
Targeted metabolomics analysis and correlation analysis. **(A)** CDCA. **(B)** DCA. **(C)** CA, **(D)** TCA, **(E)** Correlation Analysis between Differential Bacteria, BA Metabolites, and Inflammatory Factors. *P<0.05, **P<0.01, ***P<0.001.

According to the results of non-targeted metabolomics, we found that the effect of CMME on ALI is likely to be realized by influencing BA metabolism. BA metabolites in mouse plasma were qualitatively and quantitatively determined by targeted metabolomics to compare the changes of BA metabolic profile. Quantitative statistics were performed for TCA, CA, DCA and CDCA that showed significant differences in target metabolism, and the results of each group were shown in **Figure 10**. The content of four different metabolites in the Mod group was decreased compared with that in the Con group, with the difference being highly significant (P<0.001). After CMME intervention, the reduction of BA content was reversed (P<0.001), and the content of CA in CMME group was close to that in Con group (P<0.05). The content of TCA in plasma was 1781.63, 1117.10, 2350.92 nmol/mL in Con, Mod and CMME respectively, which was the highest among the four BAs. Moreover, CMME had the strongest reversing ability on TCA, and TCA was significantly higher than that in Con group (P<0.001). The content of CDCA in Con, Mod and CMME was 46.43, 15.82, 39.84 nmol/mL respectively, and the content of CDCA was the lowest among the four BAs.

According to the different bacteria genera in the analysis results of the above three groups, and combined with the serum inflammatory factor data, Spearman correlation analysis was conducted between the different bacteria genera, different BA metabolites and inflammatory factors. As depicted in **Figure 10E**, significant correlations were found between inflammatory factors levels in serum and most bacteria and metabolites. Specifically, TNF-α, IL-1β, and IL-6 exhibited strong positive correlations with *Bacteroides* and *Parabacteroides* (P<0.001), and significant negative correlations with *Lachnospiraceae_NK4A136_group*, *unclassified_Lachnospiraceae*, *Alloprevotella*, *Odoribacter*, and *Acinetobacter* (P<0.001). Analysis of inflammatory factors and BAs revealed negative correlations of TNF-α, IL-1β, and IL-6 with DCA, CA, and CDCA (P<0.001), and the correlation with TCA was weak.

Furthermore, correlations between BA metabolites and different bacterial genera were examined, revealing strong positive correlations (P<0.001) between DCA, CA, CDCA, and *Lachnospiraceae_NK4A136_group*, *unclassified_Lachnospiraceae*, *Alloprevotella, Odoribacter*, and *Acinetobacter*, and strong negative correlations with *Bacteroides* and *Parabacteroides*. Additionally, compared with the other three BAs, TCA showed a weak correlation with related bacteria. These findings underscore the close relationship between differential metabolites and bacterial genera, highlighting the intricate crosstalk between intestinal flora, metabolites, and lung injury.

## 4 Discussion

The pathogenesis of ALI generally encompasses a variety of pathological processes (20). Exogenous stimulation caused alveolar epithelial injury, leading to inflammatory cell aggregation and excessive release of inflammatory cytokines, which are central to ALI (21, 22). The degree of pulmonary edema can be assessed by the W/D ratio of lung tissue (13), with CMME observed to mitigate LPS-induced pulmonary edema, reduce inflammatory cell infiltration, and improve septal thickness to alleviate lung tissue injury. Inflammatory factors are pivotal in ALI pathogenesis, and our findings demonstrate that CMME significantly reduces TNF-α, IL-1β, and IL-6 levels in serum, BALF, and lung tissue, alongside mRNA expression in lung tissue. This aligns with previous studies reporting that phycocyanin effectively reduces inflammatory factors and improves lung injury (23). Oxidative stress is strongly associated with ALI onset (24), marked by increased oxidative stress and reduced antioxidant capacity SOD, a vital endogenous antioxidant, scavenges free radicals (25), while MDA indicates lipid peroxidation (26), higher SOD levels and lower MDA levels generally indicate reduced oxidative stress (27). CAT is an antioxidant enzyme that plays a crucial role in cellular defense. After superoxide anions are converted to hydrogen peroxide by SOD, CAT metabolizes hydrogen peroxide into water, thereby exerting its antioxidant capacity (28). ALI is also characterized by immune cell accumulation in alveoli (29), with neutrophil infiltration indicating ALI severity (30), the severity and progression of ALI may be affected by the migration of neutrophils to alveolar giant cells (31). MPO serves as a neutrophil biomarker in lung tissue (32). Our results indicated CMME’s ability to reduce LPS-induced MPO levels, preliminarily judging that CMME inhibited the recruitment of NEU. And IF results showed that CMME reduce the expression of Ly6G and CD11b in lung tissue, it further indicated that CMME could inhibit neutrophils recruitment. This is consistent with the fact that CMME reduces neutrophils in the blood. When macrophages are activated in response to a pathogen attack, they produce inflammatory factors that promote inflammation. The expression of CD68 and F4/80 in the Mod group lung tissues was significantly higher than those in the Con group, reflecting the enhanced activation of macrophages, and promoting the recruitment of neutrophils to promote inflammation, while CMME inhibited the expression (33). IF results showed that CMME inhibited the activation of inflammatory cells, supporting the potential of CMME to improve ALI pathology.

Intestinal flora plays a critical role in maintaining immune function, preventing bacterial translocation, and improving lung injury (34). Bidirectional gut-lung interactions are increasingly recognized, with intestinal flora modulation showing promise in pneumonia treatment. Studies indicate that Qing-Fei-Pai-Du and Strictosamide effectively alleviate ALI by modulating intestinal flora (35, 36). Our study similarly shows CMME restores intestinal flora diversity in LPS-induced mice, increasing *Firmicutes* abundance while decreasing *Bacteroides*, with various *Firmicutes* groups known as probiotics (13). We observed significantly elevated *Bacteroides* abundance in LPS-induced mice correlating with inflammation, positively correlated with TNF-α, IL-1β, and IL-6, this finding illustrates previous research showing *Firmicutes* negatively correlated with inflammatory factors, while *Bacteroides* exhibit a positive correlation. *Proteobacteria* levels also rises in LPS-induced mouse flora, mirroring SHEN’s findings of increased *Proteobacteria* in injured lung tissue (27), suggesting parallel changes in lung and intestinal flora (23). Spearman analysis showed a strong positive association between inflammatory factors and *Proteobacteria*, possibly due to the activation of TLR4 by *Proteobacteria* leading to an inflammatory response, this includes heightened levels of circulating IL-1β, which conveys inflammatory signals to the lungs, thereby activating NF-κB (10). Additionally, beneficial bacteria *Lachnospiraceae*, *Alloprevotella*, *Megasphaera*, and *Bifidobacterium* decreased in ALI mice, with CMME reversing these changes, potentially regulating inflammation by boosting beneficial bacteria. This result is consistent with Zhang’s findings that *Lachnospiraceae* produces butyrate to relieve inflammation (12).

The change of intestinal flora can alter the body’s metabolic phenotype, with microbiota-metabolite interactions pivotal in host performance (37). The lung senses intestinal microbial metabolites like primary and secondary BAs (38), impacting the gut-lung immune axis (39). Many studies explore drug mechanisms via the “microbe-metabolite-lung” axis, for example, cryptotanshinone has been reported to reduce lung inflammation by regulating the gut microbiota and BA metabolites in mice (40). Our metabolomic analysis identified the effects of CMME on BA, purine, and nucleotide metabolic pathways. ALI mice showed a significant increase in ATP, it has been reported that ATP is released from cells in the process of lung inflammation (41). For example, Wei’s study found that ATP expression increased when mechanical ventilation induced lung injury (42). As mentioned in Narasaiah’s report, ATP has been proved to activate inflammatory-dependent caspase-1 cleavage, thereby stimulating the secretion of IL-1β and leading to lung inflammation. In addition, the production of ROS may also be induced by ATP (43, 44). Sun believes that the anti-injury effect of quercetin are linked to purine metabolism (45). BA metabolism, closely linked to intestinal flora, drew our focus. Liver cholesterol converts to BAs, further metabolized by gut microbiota, primarily through bile salt hydrolase-active bacteria like *Bacteroides* and *Lactobacillus*. Our results showed that five BA metabolites, TCA, salicylic acid, DCA, CA and CDCA, were enriched in the BA metabolic pathway. After CMME treatment, *Bacteroides* decreased significantly, and CDCA, TCA, DCA and CA all increased. Spearman’s study showed that *Bacteroides* and *Proteobacteria* were negatively correlated with several BAs, which was consistent with the study of Zhang on the relationship between Bacteroides and CDCA and CA levels (46). Intestinal microbiota and plasma metabolomics suggests that CMME may alleviate lung injury through microbiota and BA metabolism.

At present, the role of BAs in lung diseases has also been reported. For example, it was found that bear bile powder mainly composed of tauroursodeoxycholic acid and taurochenodeoxycholic acid sodium salt could reduce MPO activity and inhibit the activation of NF-κB pathway, thus alleviating ALI (47). He et al. found that ursodeoxycholic acid can inhibit acute lung injury caused by sepsis (48), and obeticholic acid can relieve oxidative stress (49). Obeticholic acid can activate farnesoid-X receptor regulatory signaling pathway to play an anti-pneumonia role (50). In addition, ursodeoxycholic acid has been reported that it could inhibit cytokine secretion in ovalbumin sensitive mouse asthma models through farnesoid-X receptors (51), which provides evidence that BA metabolites can mediate the signaling pathway to play a role in lung inflammation.

## 5 Conclusions

In summary, our experimental findings demonstrate that CMME effectively reduces lung inflammation and improves LPS-induced ALI in mice. Our exploration of gut microbiota and metabolomics suggests that CMME’s mechanism of action in regulating LPS-induced lung inflammation may involve modulation of intestinal flora and body metabolism. However, the specific mechanisms underlying these effects warrant further investigation.

## Data Availability

The raw data supporting the conclusions of this article will be made available by the authors, without undue reservation.
