# Supplementary figures and images for: Cordyceps militaris solid medium extract alleviates lipopolysaccharide-induced acute lung injury via regulating gut microbiota and metabolism
Source: Front Immunol. 2025 Jan 20;15:1528222. doi: 10.3389/fimmu.2024.1528222 (PMC11788161; doi:10.3389/fimmu.2024.1528222)

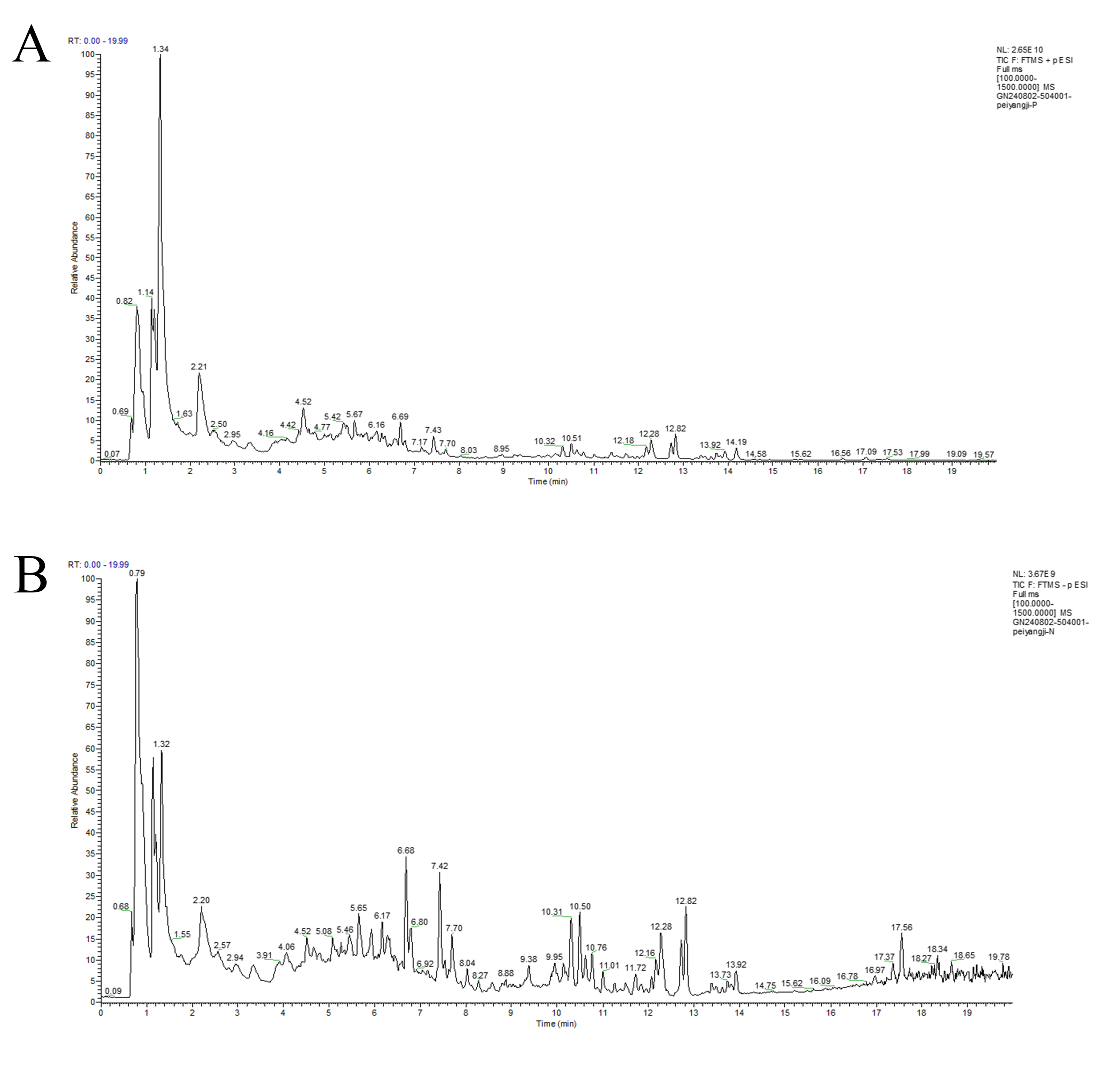

Supplement: Supplementary file 1 [file Image1.tif]
